# The Procoagulant Snake Venom Serine Protease Potentially Having a Dual, Blood Coagulation Factor V and X-Activating Activity

**DOI:** 10.3390/toxins12060358

**Published:** 2020-05-29

**Authors:** Zorica Latinović, Adrijana Leonardi, Cho Yeow Koh, R. Manjunatha Kini, Alenka Trampuš Bakija, Jože Pungerčar, Igor Križaj

**Affiliations:** 1 Department of Molecular and Biomedical Sciences, Jožef Stefan Institute, Jamova cesta 39, SI-1000 Ljubljana, Slovenia; zorica.latinovic@ijs.si (Z.L.); adrijana.leonardi@ijs.si (A.L.); joze.pungercar@ijs.si (J.P.); 2 Jožef Stefan International Postgraduate School, Jamova cesta 39, SI-1000 Ljubljana, Slovenia; 3 Protein Science Laboratory, Department of Biological Sciences, Faculty of Science, National University of Singapore, 14 Science Drive 4, Singapore 117543, Singapore; choyeow@nus.edu.sg (C.Y.K.); dbskinim@nus.edu.sg (R.M.K.); 4 Department of Medicine, Yong Loo Lin School of Medicine, National University of Singapore, 1E Kent Ridge Road, Singapore 119228, Singapore; 5 Division of Pediatrics, University Medical Center, Bohoričeva 20, SI-1000 Ljubljana, Slovenia; alenka.trampus@kclj.si

**Keywords:** FV activator, FX activator, procoagulant, snake venom, serine protease

## Abstract

A procoagulant snake venom serine protease was isolated from the venom of the nose-horned viper (*Vipera ammodytes ammodytes*). This 34 kDa glycoprotein, termed *Vaa*SP-VX, possesses five kDa N-linked carbohydrates. Amino acid sequencing showed *Vaa*SP-VX to be a chymotrypsin-like serine protease. Structurally, it is highly homologous to *Vaa*SP-6 from the same venom and to nikobin from the venom of *Vipera nikolskii*, neither of which have known functions. *Vaa*SP-VX does not affect platelets. The specific proteolysis of blood coagulation factors X and V by VaaSP-VX suggests that its blood-coagulation-inducing effect is due to its ability to activate these two blood coagulation factors, which following activation, combine to form the prothrombinase complex. *Vaa*SP-VX may thus represent the first example of a serine protease with such a dual activity, which makes it a highly suitable candidate to replace diluted Russell’s viper venom in lupus anticoagulant testing, thus achieving greater reliability of the analysis. As a blood-coagulation-promoting substance that is resistant to serpin inhibition, *Vaa*SP-VX is also interesting from the therapeutic point of view for treating patients suffering from hemophilia.

## 1. Introduction

Snakes use their venom for defense and to immobilize and digest their prey. These functions are accomplished by enzymatic and/or non-enzymatic components of the venom and are usually characterized by high target specificity and effectiveness, thermal stability, and resistance to proteolysis [1]. The venoms of *Viperidae* snakes are rich in proteins that strongly affect the hemostatic system by promoting or inhibiting platelet aggregation, blood coagulation, and fibrinolysis [2]. Members of the two families of snake venom enzymes, serine proteases (SVSPs) and metalloproteases (SVMPs), exert procoagulant activity by activating one or more coagulation factors in the blood coagulation cascade [3]. Depending on their principal target in the coagulation system, they are usually thrombin-like enzymes (TLEs), activators of prothrombin, factor V (FV), and FX. TLE and FV activators are exclusively SVSPs, while activators of FX and prothrombin can be either SVSPs or SVMPs [4].

SVSPs are abundantly present in viperid venoms, mostly as monomeric glycoproteins of 26 to 67 kDa. Their mass depends on the extent of either *N*- or *O*-glycosylation. SVSPs are classified within the clan PA as subclan S, family S1 (chymotrypsin), or subfamily A of the proteolytic enzymes [5]. Such proteases are characterized by a typical chymotrypsin fold and two six-stranded β-barrels. Their active sites lie in the cleft between the latter and include the canonical catalytic triad His-Asp-Ser [6]. Despite having a 50% to 80% shared identity of their amino acid sequences, SVSPs differ substantially in their substrate specificity, and consequently, in their pharmacological activity [7]. SVSPs that affect hemostasis express trypsin-like activity, hydrolyzing the BApNA (N_α_-benzoyl-*L*-arginine 4-nitroanilide) substrate. They are not inhibited by endogenous serine protease inhibitors (serpins) in the presence of heparin [8]. Consequently, SVSPs can be used in medical treatments and the diagnosis of blood coagulation irregularities [9]. The two best-known SVSP-based medical products are Viprinex^®^ and Defibrase^®^. Viprinex^®^ (also known as Ancrod) is a defibrinogenating agent derived from Malayan pit viper venom and has been investigated for the treatment of acute ischemic stroke [10]. Defibrase^®^ is a therapeutic product based on a procoagulant SVSP called batroxobin from *Bothrops atrox* venom. It is used to decrease fibrinogen levels, leading to the inhibition of thrombogenesis and the prevention of thrombotic diseases. Batroxobin, sold as Reptilase^®^, is also used to determine the blood clotting time (Reptilase^®^ time) [11].

The nose-horned viper (*Vipera ammodytes ammodytes*—*Vaa*), the most venomous snake in Europe, exhibits diverse hematotoxic and coagulopathic effects in vivo [12,13] and in vitro [14]. *Vaa* venom contains diverse types of enzymes, such as secreted phospholipases A_2_ (sPLA_2_s), SVMPs, L-amino acid oxidases (LAAOs), and SVSPs. Although the latter are highly abundant in the venom, they have not been the most widely studied.

Four *Vaa* SVSPs have so far been purified and characterized: two kallikrein-like enzymes [15]; a fibrin(ogen)ase with an unconventional catalytic triad, VaSP1 [16]; and an anticoagulant, which is the enzymatically inactive SVSP homolog *Vaa*SPH-1 [12]. Comprehensive transcriptomic and proteomic analysis of *Vaa* venom has revealed the existence of at least ten other SVSP molecules [17]. In this study, one of these SVSPs has been purified and characterized. *Vaa*SP-VX, as we named this molecule, is a potent procoagulant. We have shown that its blood-coagulation-promoting effect is likely due to its FV- and FX-activating activities. The activation of the precursors of both components that form the prothrombinase complex by a single venom protease would be unique and would endow *Vaa*SP-VX with promising medical potential.

## 2. Results

### 2.1. Isolation and Biochemical Characterization of VaaSP-VX

**Figure 1:**
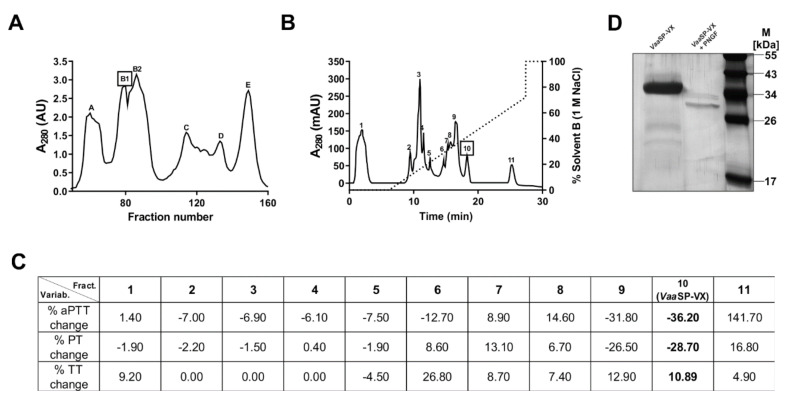
The purification and basic characterization of *Vaa*SP-VX. (**A**) Size-exclusion chromatography of the raw *Vaa* venom was carried out on a Sephacryl S-200 superfine column. The elution of proteins was monitored at 280 nm (A_280_). (**B**) Fraction B1 from the size-exclusion chromatography was sub-fractionated on a Mono S 5/50 GL ion-exchange fast protein liquid chromatography (FPLC) column. The retained proteins were eluted using a linear gradient of NaCl (dotted line) in 11 fractions. (**C**) Fractions were tested for their effect on blood coagulation. Three clinical blood coagulation assays were used: a prothrombin time assay (PT), an activated partial thromboplastin time assay (aPTT), and a thrombin time assay (TT). The table shows the effects of each fraction on aPTT, PT, and TT in terms of the percent change relative to the value obtained in the control experiment performed without the addition of a fraction. Fraction 10, which displayed a pronounced procoagulant effect, contained pure *Vaa*SP-VX (values in bold). (**D**) Under reducing conditions, SDS-PAGE electrophoresis revealed *Vaa*SP-VX to be a monomeric protein of about 34 kDa. Incubation of *Vaa*SP-VX with peptide N-glycosidase F (PNGF) at 37 °C overnight followed by SDS-PAGE analysis displayed a lower molecular mass of about 29 kDa. *Vaa*SP-VX was thus found to be an N-glycosylated protein.

**Figure 2:**
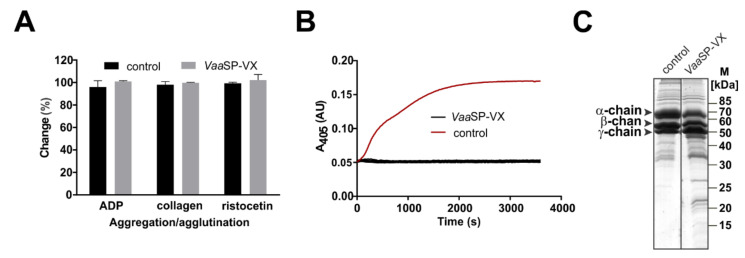
Effects of *Vaa*SP-VX on platelets and fibrinogen. (**A**) Platelet-rich plasma was pre-incubated with 1 μM *Vaa*SP-VX at 37 °C. The agonists of platelet aggregation (ADP and collagen) or agglutination (ristocetin) were then added. Platelet aggregation/agglutination was followed by monitoring the optical density of the samples. Control values, obtained in the absence of the venom protein, were regarded as a 100% change of optical density. Evidently, *Vaa*SP-VX did not affect the platelet function. (**B**) *Vaa*SP-VX, in concentrations from 0.1 to 10 µM, was added to human fibrinogen. Thrombin was used as a positive control. The formation of fibrin clots was tracked using continuous measurements of the absorbance at 405 nm (A_405_). In the control experiment, thrombin induced a transformation of fibrinogen to fibrin, as indicated by the rise in A_405_ (red line). For all concentrations tested, *Vaa*SP-VX did not stimulate clotting (black lines). (**C**) Human fibrinogen, consisting of α, β, and γ chains, was incubated with *Vaa*SP-VX in a mass ratio of 100:1. In the control experiment, no *Vaa*SP-VX was added. Following incubation at 37 °C for 60 min, the samples were analyzed on SDS-PAGE under reducing conditions and stained with PageBlue^®^. Only a slight degradation of the α-chain was apparent; therefore, the pronounced procoagulant effect of *Vaa*SP-VX could not be due to its action on fibrinogen.

*Vaa* venom was size-fractionated into several protein peaks using gel filtration chromatography on a Sephacryl S-200 Superfine (Figure 1A). The B1 fraction, which contained proteases with molecular masses in the range of 30 to 40 kDa, was further separated using cation-exchange chromatography (Figure 1B). Eleven peaks were collected, dialyzed against buffer B, and tested for their effect on blood plasma coagulation. They exhibited either pro- or anti-coagulant activity (Figure 1C). We focused our attention on peak 10, which displayed the most pronounced procoagulant effect by shortening the prothrombin time assay (PT) and the activated partial thromboplastin time assay (aPTT). At the same time, this fraction did not influence the ADP- or collagen-induced aggregation, or ristocetin-induced agglutination of platelets (Figure 2A). As established using SDS-PAGE analysis and Edman sequencing, the fraction was homogenous, containing an SVSP with the N-terminal amino acid sequence VIGGDECNINEHPFL. This procoagulant molecule was termed *Vaa*SP-VX. From 1 g of the raw *Vaa* venom, we obtained 1 mg of pure *Vaa*SP-VX, indicating a purification yield of 0.1%.

*Vaa*SP-VX is an *N*-glycosylated protein of about 34 kDa (Figure 1D). When treated with peptide N-glycosidase F (PNGF), which cleaves off a glycan moiety from the *N*-glycosylated proteins, its molecular mass dropped by about 5 kDa. Typical of glycosylated proteins, *Vaa*SP-VX was present in seven glycoforms, with isoelectric points (pIs) between 6 and 10 (not shown). The main glycoform was neutral, displaying a pI of approximately 7.

### 2.2. VaaSP-VX Did Not Act Like Thrombin

To explain the molecular background of the pronounced procoagulant effect of *Vaa*SP-VX, we examined whether *Vaa*SP-VX acts like thrombin, i.e., whether it can clot fibrinogen directly. The specific blood coagulation assay, the thrombin time assay (TT), which would confirm the thrombin-like activity, showed only a slightly extended value of 10.89 (±5.02 %) in the presence of 1 μM *Vaa*SP-VX (Figure 1C). In accordance with this, *Vaa*SP-VX also failed to convert fibrinogen into a stable fibrin clot (Figure 2B). These results are consistent with the low fibrinogenolytic activity of the enzyme. As demonstrated using the SDS-PAGE analysis (Figure 2C), it only partially cleaved the α chain of fibrinogen, while the other two fibrinogen chains remained intact. It may thus be concluded that the acceleration of blood coagulation by *Vaa*SP-VX was due to its action on blood coagulation factors in the coagulation cascade upstream of thrombin.

### 2.3. Activity of VaaSP-VX in Fresh Human Plasma

**Figure 3:**
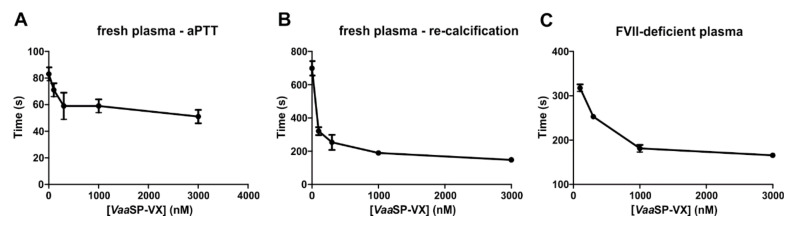
Clotting of fresh or coagulation factor-depleted plasmas in the presence of *Vaa*SP-VX. (**A**) To fresh human plasma, *Vaa*SP-VX was added at indicated concentrations and incubated at 37 °C for 10 min. The activated partial thromboplastin (aPTT) was then measured as described under Section 5: Materials and Methods. (**B**) To assess the plasma re-calcification time, the same procedure as in (**A**) was used, except the addition of the aPTT SA reagent was replaced by the addition of PBS. (**C**) To human plasma depleted in FVII, *Vaa*SP-VX was added at the indicated concentrations. After 2 min of incubation at 37 °C, 25 mM CaCl_2_ was added and the formation of fibrin clotting was observed by measuring the absorbance at 405 nm (A_405_).

The addition of *Vaa*SP-VX to fresh human plasma resulted in a dose-dependent shortening of aPTT and the re-calcification time compared with those of the control (Figure 3A,B). This indicated that *Vaa*SP-VX activated one or more of the coagulation factors. Since PT, which reflects perturbations in the extrinsic pathway of blood coagulation, was shortened to almost the same extent as aPTT, which points to abnormalities in the intrinsic pathway of blood coagulation, we concluded that *Vaa*SP-VX most probably interfered with blood coagulation somewhere in the cascade common to both pathways in the so-called common pathway of blood coagulation. This involved the prothrombinase complex, prothrombin, and fibrinogen. The prothrombinase complex is an assembly of activated FV and FX (FVa and FXa) on the negatively charged phospholipid membrane of platelets. Before proteolytic activation, FV and FX exist as soluble proteins in the blood. The prothrombinase complex activates prothrombin to thrombin, which then converts soluble fibrinogen to form an insoluble fibrin clot.

### 2.4. VaaSP-VX-Induced Clotting of Plasma Deficient in FVII

Commercial plasmas deficient in FVII or FIX only coagulate in the presence of Ca^2+^ ions if supplemented by phospholipids to enable the establishment of coagulation complexes, extrinsic or intrinsic tenase, and a prothrombinase complex, in which one or the other tenase complexes responsible for activation of FX is not present. Alternatively, such plasmas would coagulate only if *Vaa*SP-VX were to activate FX directly or would act as FXa. Indeed, Ca^2+^-containing FVII-deficient plasma clotted in the presence of *Vaa*SP-VX but remained unaffected if PBS alone was added (negative control). For example, 100 nM *Vaa*SP-VX induced clotting of FVII-deficient plasma in 320 s. The effect was dose-dependent: the more *Vaa*SP-VX that was added the quicker the clotting (Figure 3C). Such a result has led us to the conclusion that *Vaa*SP-VX induced plasma clotting by stimulating the conversion of prothrombin to thrombin, either by activating FX to FXa or by acting in the same way as FXa.

### 2.5. VaaSP-VX Did Not Activate Prothrombin Directly

To test the hypothesis that *Vaa*SP-VX induces a procoagulant effect by mimicking the action of FXa, we incubated prothrombin with *Vaa*SP-VX. The formation of α-thrombin was probed using α-thrombin-specific fluorogenic substrate Boc-Val-Pro-Arg-AFC, whose hydrolysis was inspected by measuring the fluorescence at 500 nm. The substrate remained untouched; therefore, we concluded that *Vaa*SP-VX was unable to activate prothrombin to α-thrombin. In agreement, *Vaa*SP-VX was also not able to hydrolyze a chromogenic substrate (S-2222^TM^) that contains the FXa-cleavage site of prothrombin.

### 2.6. VaaSP-VX Activated FX and Likely Also FV

**Figure 4:**
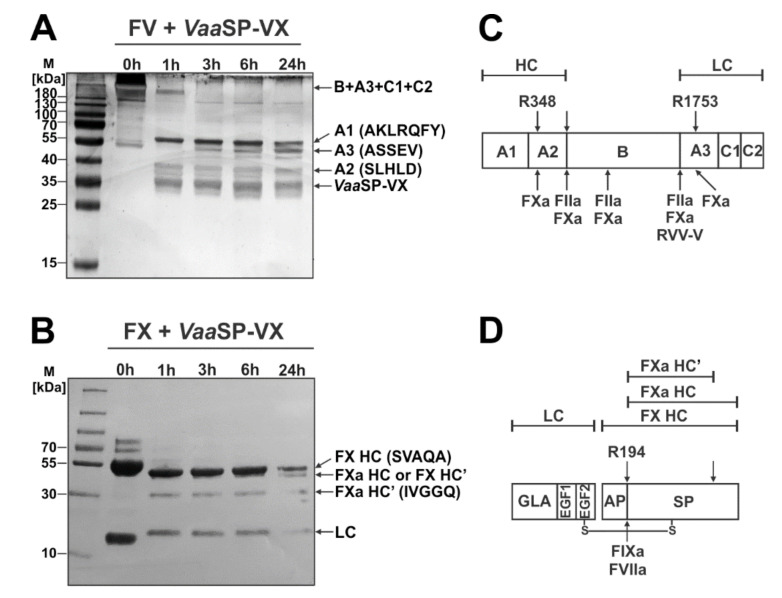
Hydrolysis of FV and FX by *Vaa*SP-VX. Bovine FV (**A**), (or human FX (**B**)), was incubated with or without *Vaa*SP-VX. An aliquot of the respective reaction mixture (10 µL) was taken immediately (0 h) and then after 1, 3, 6, and 24 h of incubation at 37 °C; it was then analyzed on SDS-PAGE under reducing conditions. The gels were stained with PageBlue^®^ to visualize the protein bands. As products of degradation by *Vaa*SP-VX, the protein bands of FV or FX (arrows) were electroblotted to the PVDF membrane and N-terminally sequenced to define the cleavage positions. *Vaa*SP-VX activated FV into FVa and FX into FXa. Schematic presentations of the domain structures of FV (**C**) and FX (**D**), with the indicated sites of activation by the following physiological activators: FXa, thrombin (FIIa), FVIIa, and FIXa, and RVV-V (below), and *Vaa*SP-VX (above). Abbreviations: A1, A2, A3, B, C1, and C2: structural domains of FV; AP: activation peptide; EGF, GLA, and SP: epidermal growth factor-like, γ-carboxyglutamic acid-rich, and serine protease domain, respectively; LC, HC, and HC’: light, heavy, and C-terminally truncated heavy chain, respectively.

Next, we tested the hypothesis that *Vaa*SP-VX induces the procoagulant effect by promoting the formation (and thus the activity) of the prothrombinase complex, i.e., by the activation of FX or/and FV. As is evident from Figure 4, *Vaa*SP-VX was able to cleave both FV and FX. To verify whether the observed cleavages were converting these factors to their activated forms, we blotted the fragments from SDS-PAGE gels to polyvinylidene difluoride (PVDF) membranes and sequenced them.

*Vaa*SP-VX initially cleaved bovine FV (in the first hour of incubation) into three main fragments with apparent molecular masses of 38, 55, and about 250 kDa (Figure 4A). The N-terminal sequence SLHLD of the 38 kDa fragment corresponded to part of the A2 domain of the bovine FVa. This fragment was excised from FV by two cleavages, after Arg^348^ within the A2 domain and at the N-terminus of the B domain (Figure 4C). The 55 kDa fragment, with an N-terminal sequence of AKLRQFY, corresponding to the FV domain A1, cleaved off FV at Arg^348^ (Figure 4C). The largest fragment represented the rest of the FV molecule. It consisted of domains B, A3, C1, and C2 (Figure 4A,C). The fragment at about 50 kDa (Figure 4A) accumulated over time, with the N-terminal sequence ASSEV corresponding to the N-terminus of the light chain (LC) of FVa. This was a result of the sub-cleavage of the 250 kDa fragment after Arg^1753^ within the A3 domain. *Vaa*SP-VX thus cleaved bovine FV at the C-terminal end of Arg^348^ and Arg^1753^, i.e., at the same sites as its natural activator FXa [18,19,20].

The result of the incubation of human FX with *Vaa*SP-VX were two main protein fragments, one at 36 kDa and the other at 30 kDa (Figure 4B). The band at 42 kDa corresponded to the heavy chain (HC) of FX, while the band at 19 kDa corresponded to its LC, as we confirmed using Edman sequencing. We assumed that the band at 36 kDa was the FXa HC, the product of the removal of part the activation peptide (AP) from the N-terminus of the FX HC. The FXa HC was then further trimmed by *Vaa*SP-VX somewhere at its C-terminal side to give the product FXa HC’, which was 30 kDa in mass. The FXa HC’ had the N-terminal sequence IVGGQ, which was the sequence of the FXa HC resulting from the cleavage of the FX HC after Arg^194^ by physiological activators of FX, FVIIa, and FIXa (Figure 4D). Consistently, as FVIIa, *Vaa*SP-VX hydrolyzed S-2288^TM^. However, the chromogenic substrates of FIXa and FXa were not cleaved by *Vaa*SP-VX, offering us the possibility to test its FIX- and FX-activating abilities. Pre-incubation of FX with *Vaa*SP-VX resulted in the hydrolysis of S-2222^TM^, which is the specific substrate of FXa. *Vaa*SP-VX thus converted human FX into its active form, confirming the conclusion suggested by the SDS-PAGE analysis.

### 2.7. Amino Acid Sequence of VaaSP-VX

**Figure 5:**
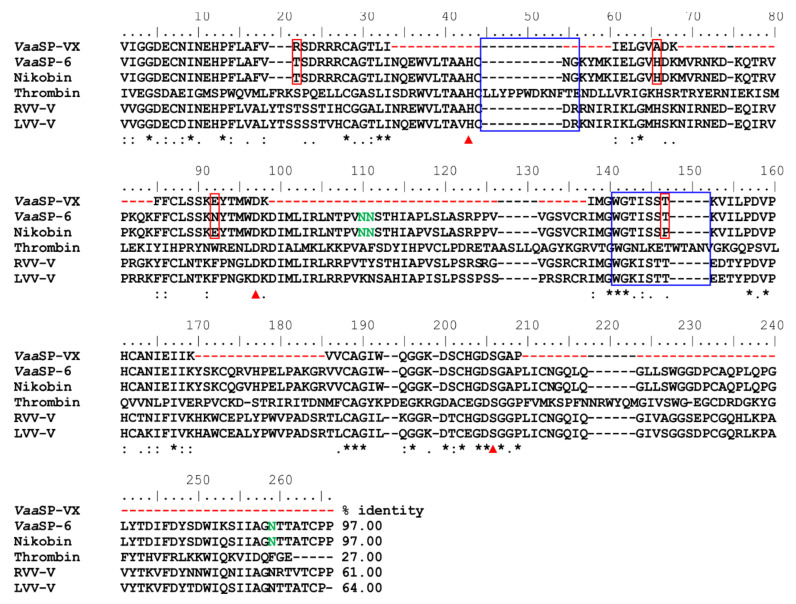
Partial amino acid sequence of *Vaa*SP-VX and its comparison with sequences of some relevant serine proteases (SPs). *Vaa*SP-VX was sequenced using a combination of Edman degradation and tandem MS. Based on its established partial structure, *Vaa*SP-VX was recognized as an isoform of *Vaa*SP-6, whose complete cDNA sequence was determined and deposited at the National Center for Biotechnology Information (NCBI, MG958495). Nikobin, an SP isolated from *Vipera nikolskii* venom, is a protein with the same extent of structural identity to the sequenced parts of *Vaa*SP-VX as *Vaa*SP-6 (97.0%). Amino acid residues, where differences between *Vaa*SP-VX and *Vaa*SP-6 or nikobin occurred, are in red boxes. Red arrows at the bottom of the aligned sequences point to three amino acid residues that constituted the catalytic triad in SPs (His57, Asp102, and Ser195, according to chymotrypsin numbering). The undetermined parts of the *Vaa*SP-VX sequence are denoted by red dashes, while predicted gaps are denoted by black ones. Amino acid residues that may be *N*-glycosylated in *Vaa*SP-VX (if present) are printed in green. Other SPs that were aligned are human thrombin, which is the main physiological activator of FV, and two snake venom FV activators, namely RVV-V from *Daboia siamensis* and LVV-V from *Macrovipera lebetina*. Except for thrombin, the two loops, 44-loop and 148-loop (in blue squares), are absent in all presented FV activators from snake venoms. This was established as the reason why snake venom SPs were resistant to inhibition by antithrombin. Below the sequences, an asterisk (*) designates a position that is strictly conserved in all aligned sequences, a colon (:) denotes a position in which only conservative substitutions were detected, and a dot (.) denotes a position harboring semi-conservative substitutions. On all other sites, the substitutions were non-conservative. The percentages of identity between the sequence of *Vaa*SP-VX and the corresponding sequences in *Vaa*SP-6, nikobin, thrombin, RVV-V, and LVV-V are presented.

Proteolysis of the purified *Vaa*SP-VX and MS/MS analysis of the resulting peptides led to the establishment of 40% of the amino acid sequence (Figure 5). Using this sequence, we screened the non-redundant National Center for Biotechnology Information (NCBI) database and the in house *Vaa* venom gland cDNA sequence database. No complete match was found; therefore, we continued screening the cDNA library, using a PCR primer based on the N-terminal sequence of *Vaa*SP-VX. As the search was not successful, a less-specific PCR primer was used directed toward cDNAs of all currently known SVSPs in the *Vaa* venom gland cDNA library [17]. In this way, a cDNA (MG958495), encoding *Vaa*SP-6, which is a protein almost identical to *Vaa*SP-VX in the known parts of its structure, was isolated (Figure 5). In the aligned regions, *Vaa*SP-VX and the predicted mature form of *Vaa*SP-6 differ in only 3 of the 100 amino acid residues: Arg^22^, Ala^66^, and Glu^92^ in *Vaa*SP-VX are replaced by Thr, His, and Asn in *Vaa*SP-6. *Vaa*SP-VX and *Vaa*SP-6 may thus be considered to be isoforms. Searching in the non-redundant NCBI database revealed another SVSP with a high sequence similarity to that of *Vaa*SP-VX, namely nikobin, from the venom of *Vipera nikolskii* [21]. The predicted amino acid sequence of the mature form of nikobin shared 97.42% identity with *Vaa*SP-6 and 97.00% with the sequenced parts of *Vaa*SP-VX (Figure 5). Three potential *N*-glycosylation sites were conserved in *Vaa*SP-6 and nikobin (Asn^110^, Asn^111^, and Asn^259^). Being located within sequences not covered by our MS/MS results, the existence of these sites was not confirmed in *Vaa*SP-VX. *Vaa*SP-6 potentially contained an extra *N*-glycosylation site at Asn^92^, which was otherwise absent in *Vaa*SP-VX and nikobin due to the Asn/Glu substitution.

## 3. Discussion

*Vaa* venom is a rich source of proteins that act on the hemostatic system [14]. In the present study, we purified and characterized a procoagulant SP from this venom and named it *Vaa*SP-VX.

*Vaa*SP-VX was isolated in a two-step chromatographic procedure, in which gel filtration was followed by cation-exchange chromatography (Figure 1). *Vaa*SP-VX was found in the fraction, which substantially shortened aPTT and PT without interfering with the platelet function (Figure 1C and Figure 2A).

*Vaa*SP-VX was found to be a single-chain *N*-glycosylated protein with an apparent molecular mass of 34 kDa. The mass of the glycan moiety was about 5 kDa (Figure 1D), representing approximately 15% of the total mass of the molecule. It was demonstrated that glycosylation affected the enzymatic activity and specificity of SVSPs by defining target recognition and by binding inhibitors [22,23,24,25]. Therefore, it may be expected that carbohydrates in *Vaa*SP-VX have similar functions.

The partial amino acid sequence of *Vaa*SP-VX was determined using Edman and MS/MS sequencing. The sequenced part of *Vaa*SP-VX, which covered about 40% of the predicted mature protein, exhibited a high sequence identity with another SVSP from the *Vaa* venom, namely *Vaa*SP-6 (MG958495), with unknown functions. These two proteins differed in the sequenced parts by only three amino acid residues, and therefore, could be regarded as isoforms (Figure 5). This conclusion is supported by a 2D-PAGE-based proteomic study of the *Vaa* venom [17], in which *Vaa*SP-6 isoforms were identified in 21 spots positioned in the pI range from 6 to 9. Moreover, in 3 of these 21 spots, a peptide ^92^EYTMWDKDIMLIR^104^ was detected, which was identified as a part of nikobin, which is an SVSP from the closely related snake *V. berus nikolskii* (E5AJX2) (Figure 5), but may also have originated from *Vaa*SP-VX. The first part of this peptide, ^92^EYTMWDK^98^, which was only identified in the case of *Vaa*SP-VX, was the same in the two molecules, as was the sequence preceding it, namely ^85^FFCLSSK^91^. Nikobin and *Vaa*SP-VX were therefore identical in the region ^85^FFCLSSKEYTMWDK^98^, while *Vaa*SP-6 was distinguished in this part from the other two SPs by the unique ^92^E/N substitution. In the non-redundant NCBI database, nikobin was the most similar protein to *Vaa*SP-VX and *Vaa*SP-6, displaying a 97% sequence identity (Figure 5). However, some *Vaa*SPs may differ just in their glycosylation patterns, representing glycoforms of identical amino acid sequences.

The comparable effects of *Vaa*SP-VX on aPTT and PT (Figure 1C) and clotting of FVII-deficient plasma by *Vaa*SP-VX (Figure 3C) suggested that the action of this venom protease was directed toward the common pathway of the blood coagulation, in which FXa, as a component of the prothrombinase complex, activates prothrombin to thrombin, which then cleaves fibrinogen to form a fibrin clot [26]. We have shown that VaaSP-VX did not have the same effect as thrombin because it could not directly induce the clotting of fibrinogen (Figure 2B). Accordingly, the TT was also not shortened in its presence (Figure 1C), but moreover, it was even slightly extended. Such an effect may be explained by the defibrinogenating effect of *Vaa*SP-VX due to its partial cleavage of the α chain of fibrinogen (Figure 2C). Since we showed that *Vaa*SP-VX also did not possess FXa-like activity, its effect on blood plasma must be associated with the activity of the prothrombinase complex assembled from the activated forms of FV and FX. This suggested that *Vaa*SP-VX was involved in the activation of these blood-clotting factors. To test this hypothesis, we treated FV and FX with *Vaa*SP-VX and analyzed the cleavage products.

FV circulates in the blood as a 330 kDa protein comprised of six domains, A1, A2, A3, B, C1, and C2. In the process of its physiological activation, it is cleaved by thrombin to release the B domain. A heterodimeric FV is formed, in which the A1 and A2 domains constitute the HC and A3 and the C1 and C2 domains the LC, respectively [27]. This form of FV is further trimmed by FXa or by thrombin (FIIa). FXa cleaves FV at Arg^348^ in the A2 domain and at Arg^1753^ in the A3 domain, which produces its fully activated form (Figure 4C) [18,19,20]. Bovine FV was incubated with *Vaa*SP-VX and the degradation products were N-terminally sequenced. In this way, we established that *Vaa*SP-VX cleaved FV at the same positions as FXa did. The report that treatment of human FV with FXa induced the FVa activity, which is associated with the cleavage at Arg^1765^ (this is Arg^1753^ in bovine FV) [28], strongly suggests that *Vaa*SP-VX could convert not only bovine but also human FV into its activated form (Figure 4A,C). FV has already been identified as a target of SVSPs. Two well-studied examples are the FV activators from the venoms of *Daboia* snakes, 26 kDa RVV-V from *D. siamensis* and 28.4 kDa LVV-V from *V. (M.) lebetina* [29,30]. However, they activate human FV in a thrombin-like manner, cleaving it at Arg^1545^ (which corresponds to Arg^1536^ in bovine FV; Figure 4C) [31]. Both enzymes are single-chain glycoproteins. Regarding *Vaa*SP-VX, RVV-V appears in several isoforms [29]. Two of them, which have been completely sequenced, differ from each other only by six amino acid residues.

Mature FX is a vitamin-K-dependent SP zymogen, which circulates in the blood as a disulphide-linked two-chain molecule of 59 kDa [32]. It is activated by FIXa or FVIIa, which are enzymatic components of the intrinsic or extrinsic tenase complex. In both cases, FXa is produced by cleavage of the Arg^194^–Ile^195^ peptide bond in the HC of FX. As we demonstrated, *Vaa*SP-VX cleaved FX in the same way as both physiological activators (Figure 4B,D), and thus likely converted it into its activated form. Such an assumption was then also confirmed by the results of experiments using a specific chromogenic substrate of FXa. A variety of FX activators have already been found in snake venoms [4]. While FX activators from *Viperidae* venoms are mainly SVMPs, FX-activating SVSPs have only been described so far in two *Elapidae* venoms [33,34]. Like *Vaa*SP-VX, they are single-chain glycoproteins but with a much higher molecular mass (62 and 70 kDa). No structural data are available for any of them; therefore, the first structural data of FX-activating SVSP is provided in this paper. The procoagulant activity of *Vaa*SP-VX was thus the consequence of the simultaneous activation of FV and FX in a physiological manner by this venom enzyme.

The activity of blood coagulation factors is tightly regulated by endogenous protein inhibitors (serpins), such as antithrombin [35]. The serpin interaction site in thrombin includes its 44-loop and 148-loop (Figure 5). As was experimentally demonstrated using RVV-V and LVV-V [31], the absence of these two loops renders SVSPs resistant to serpins. *Vaa*SP-VX also lacks these two loops; therefore, it is expected not to be inhibited by serpins. As an activator of multiple blood coagulation factors that is resistant to inhibition by serpins, *Vaa*SP-VX is of potential interest regarding the development of an innovative clot-promoting drug for external use in patients suffering from hemophilia or other bleeding conditions [36].

Additionally, the potential double-activating activity of *Vaa*SP-VX offers an elegant solution to replacing the unreliable diluted Russell‘s viper venom (dRVV) in clinical testing of the lupus anticoagulant (LA) [37]. Two components of dRVV are important for this test: RVV-V, which is an SVSP that activates FV and RVV-X, which is an SVMP that activates FX. However, the problem with the dRVV reagent is its consistency since it is obtained from a natural source that is subject to considerable biological variations. Accordingly, the results of the LA test are frequently inconsistent. To improve the sensitivity and specificity of the test, the use of purified snake venom components for the activation of FV and FX has already been suggested [38]. An even better solution would be to use *Vaa*SP-VX, which would simultaneously activate both FV and FX.

## 4. Conclusions

We have purified and characterized the procoagulant SVSP from the venom of the nose-horned viper. This molecule, *Vaa*SP-VX, seems to be unique in terms of its ability to activate FV and FX at the same time. As suggested by its structure, it also escapes inhibition by serpins. Such traits make *Vaa*SP-VX medically interesting for replacing dRVV in LA testing and for developing novel blood-clot-promoting drugs.

## 5. Materials and Methods

### 5.1. Purification of VaaSP-VX

Crude *Vaa* venom was provided by the Institute of Immunology, Zagreb, Croatia. Lyophilized venom was kept at −20 °C. Before the analysis, it was dissolved in 50 mM Tris, 2 mM CaCl_2_, 300 mM NaCl, pH 7.0 (buffer A). Fifteen milliliters of the crude venom solution (i.e., 1 g of the venom) was applied to a Sephacryl S-200 Superfine column (100 cm × 4 cm) (GE Healthcare BioSciences AB, Uppsala, Sweden). Gel chromatography was performed at a constant flow rate of 33.6 mL/h. The concentration of proteins in the mobile phase was monitored by measuring their absorbance at 280 nm (A_280_). The B1 fraction was dialyzed against 20 mM MES, 2 mM CaCl_2_, pH 6.5 (buffer B), and further fractionated on a Mono S 5/50 GL column of the FPLC system (ÄKTA, GE Healthcare Life-Science, Uppsala, Sweden), equilibrated with the same buffer. The column-retained material was eluted using a linear gradient of NaCl (0 to 1 M) in the buffer B. *Vaa*SP-VX was eluted from the column, concentrated, and dialyzed against buffer B, at pH 7.5.

### 5.2. Polyacrylamide Gel Electrophoreses

One-dimensional polyacrylamide gel electrophoresis (PAGE) of the samples (as specified in the descriptions of figures showing the results) was performed under non-denaturing or denaturing conditions, i.e., in the absence and presence of the detergent sodium dodecyl sulphate (SDS-PAGE). For SDS-PAGE analysis, 12.5% (*m*/*v*) PA gels were used under non-reducing and reducing conditions. The proteins in gels were visualized via silver staining using the modified method of Morrissey, where protein fixation is accomplished by a mixture of water, ethanol (30% (*v*/*v*)), and acetic acid (10% (*v*/*v*)) [39]. The molecular mass standards were from Fermentas (Thermo Fisher Scientific, Vilnius, Lithuania).

Two-dimensional gel electrophoresis (2DE) of 9 μg of *Vaa*SP-VX was performed as specified in Latinović et al. [39].

### 5.3. N-Terminal Amino Acid Sequencing and Mass Spectrometry

N-terminal amino acid sequencing of the isolated *Vaa*SP-VX was performed using automated Edman degradation on a Procise 492A Sequencing System (Applied Biosystems, Foster City, CA, USA).

For the mass spectrometry (MS) analysis, 2 μg of vacuum-dried *Vaa*SP-VX was dissolved in 10 μL of 50 mM NH_4_HCO_3_ supplemented with 6 M urea. The protein was reduced via the addition of 0.25 µL of 100 mM dithiothreitol (DTT) in 50 mM NH_4_HCO_3_ and incubation at 37 °C for 1 h. The mixture was then diluted with 10 µL of 50 mM NH_4_HCO_3_ and alkylated with 0.8 µL of 250 mM iodoacetamide in 50 mM NH_4_HCO_3_ at room temperature (RT) for 45 min in the dark. Alkylation was stopped by adding 2.5 µL of DTT and then 50 mM NH_4_HCO_3_ to 90 µL. Fragmentation of the reduced and alkylated *Vaa*SP-VX was accomplished by adding 0.1 μg of endoproteinase Lys-C and standing at 37 °C overnight. Fifty nanograms of trypsin in 10 µL of 50 mM NH_4_HCO_3_ was then added, and the mixture was left overnight for further fragmentation at 37 °C. Proteolysis was stopped via the addition of formic acid to the sample to a final concentration of 0.1% (*v*/*v*). The sample was desalted and concentrated in Vivapure C18 micro spin columns (Sartorius Stedim Biotech, Göttingen, Germany) according to the manufacturer’s instructions, with formic acid replacing trifluoroacetic acid. The *Vaa*SP-VX hydrolysate was analyzed on an ion trap mass spectrometer 1200 series HPLC-Chip-LC/MSD Trap XCT Ultra (Agilent Technologies, Waldbronn, Germany), as described in Leonardi et al. [40]. MS and tandem MS (MS/MS) spectra were searched against the *Vaa* venom gland cDNA transcripts library and the non-redundant NCBI (National Center for Biotechnology Information) database using the Spectrum Mill software (A.03.03.084, Agilent Technologies, Santa Clara, CA, USA).

### 5.4. Search for the mRNA Transcript of VaaSP-VX

The cDNA sequence of *Vaa*SP-VX was searched for using the *Vaa* venom gland cDNA library of mRNA transcripts [41], and PCR primers designed using two different approaches. First, we designed the sense primer deduced from an amino acid stretch from the N-terminal region of *Vaa*SP-VX and determined using Edman degradation (CNINEHPF; cacgaattcATGTAACATAAATGAACATCCTTTC, where the additional *Eco*RI restriction site is underlined). Second, we designed a sense primer with a broader specificity (CNINEHP; cacgaattcATGTAACATAAATGAACATCCTTT). The antisense primer was the same in both cases and was deduced from the end of the coding region of other known *Vaa*SP mRNAs, including the stop codon and a part of the 5′ UTR region (tataagcTTTTCAAAAGTTTTCACGGGG, with an additional *Hin*dIII site underlined and a stop codon doubly underlined). In an attempt to obtain more specific binding of primers to template DNA, we employed different PCR protocols by using Taq or Q5 polymerase, changing the number of cycles and Tm, and lowering concentrations of primers and dNTPs.

### 5.5. N-Deglycosylation

Four micrograms of dry *Vaa*SP-VX were dissolved in 10 μL of 1% (*m*/*v*) SDS and incubated at 100 °C for 5 min. The solution was mixed with 34 μL of 1.5% (*m*/*v*) CHAPS in 50 mM Na_2_HPO_4_, pH 7.5. A total of 3 U (3 µL) of the peptide N-glycosidase F (PNGF, Roche, Mannheim, Germany) was added to half of this sample. To the other half, just water was added and served as the control. After overnight incubation at 37 °C, both samples were analyzed using SDS-PAGE on a 12.5% gel under reducing conditions. The gel was stained with colloidal silver, as described above.

### 5.6. Prothrombin Time, Activated Partial Thromboplastin Time, Thrombin Time, and Re-Calcification Time Measurements

To 45 µL of normal human plasma (Diagnostica stago, Asnières-sur-Seine, France) in a cuvette, we added an aliquot of each fraction eluted from a Mono S column in 5 µL (volume adjustment was accomplished using buffer B) to obtain the final concentration of protein in the assay of 1 µM. In the control experiment, just 5 µL of buffer B was added to human plasma. Samples were incubated for 1 min at 37 °C. Then, the prothrombin time (PT), activated partial thromboplastin time (aPTT), and thrombin time (TT) were measured using the BFT II Coagulation Analyzer (Siemens, Munich, Germany). The measurement of PT was initiated by adding 100 µL of Thromborel S reagent (lyophilized human placental thromboplastin (≤60 g/L) and CaCl_2_ (approx. 1.5 g/L); Siemens, Munich, Germany). The measurement of aPTT was initiated by adding 50 µL of Pathromtin SL reagent (SiO_2_ particles (1.2 g/L), plant phospholipids (0.25 g/L), NaCl, HEPES, pH 7.6; Siemens, Munich, Germany). After 2 min of incubation, 50 µL of 25 mM CaCl_2_ was added and the clotting time was recorded as the aPTT. The addition of 100 µL of Thrombin reagent (source of α-thrombin; Siemens, Germany) to the sample induced the clotting of plasma in a time registered as the TT.

The dependence of aPTT on the concentration of *Vaa*SP-VX was also measured in an alternative way. Fresh human plasma, aPTT soluble activator (SA) reagent (Helena Laboratories, Beaumont, TX, USA), 25 mM CaCl_2_ (Helena Laboratories, USA), and solutions of *Vaa*SP-VX in phosphate-buffered saline (PBS: 10 mM Na_2_HPO_4_, 1.8 mM KH_2_PO_4_, 137 mM NaCl, 2.7 mM KCl, pH 7.4) were pre-warmed at 37 °C. Different amounts of *Vaa*SP-VX were added to the plasma, which was incubated for 10 min at 37 °C. The aPTT SA reagent (0.1 mM Ellagic acid with a suspension of phospholipids extracted from dehydrated rabbit brain) was then added and the mixture was incubated for another 5 min. Finally, the clotting of plasma was initiated by the addition of CaCl_2_. All solutions were always added in 30 µL volumes. The formation of the clot was followed by measuring the absorbance at 405 nm (A_405_) on an Infinite M200 microplate reader (Tecan, Männedorf, Switzerland).

The same procedure, except substituting aPTT SA reagent with PBS (i.e., the procedure without supplement of phospholipids), was used to assess the full plasma re-calcification time.

The blood was donated by healthy human volunteers according to permission no. 53/08/11 of the National Medical Ethics Committee of the Republic of Slovenia.

### 5.7. Platelet Aggregation/Agglutination Assays

Platelet-rich plasma (PRP) and platelet-poor plasma (PPP) were prepared as described in Leonardi et al. [42]. To 250 μL of PRP, 25 μL of *Vaa*SP-VX solution was added, resulting in a final concentration of 1 µM *Vaa*SP-VX; it was then pre-incubated for 5 min at 37 °C. In the control experiment, 25 μL of water was added to 250 μL of PRP. Twenty-five microliters of collagen solution (DiaMed, Cressier,, Switzerland), giving a final concentration of 10 μg/mL; ristocetin (Chrono-Log Corporation, Havertown, PA, USA), resulting in a final concentration of 1 mg/mL; or ADP (Chrono-Log Corporation, USA) at a final concentration of 10 μM, were lastly added and the change in the optical density was monitored for 5 min using a Model 700 Whole Blood/Optical Lumi-Aggregometer (Chrono-Log Corporation, Havertown, PA, USA). The cuvette containing PRP corresponded to 0% light transmission and the cuvette with PPP to 100% light transmission. Blood was donated by healthy human volunteers according to permission no. 53/08/11 of the National Medical Ethics Committee of the Republic of Slovenia.

### 5.8. FVIIa, FIXa, and FXa-like Activity Testing

To test whether *Vaa*SP-VX acts in the same way as FVIIa, FIXa, or FXa, we used the following specific chromogenic substrates: S-2288^TM^ (Diapharma, West Chester Township, OH, USA) for FVIIa, Spectrozyme^®^ (American Diagnostics Inc., New York, NY, USA) for FIXa, and S-2765^TM^ and S-2222^TM^ (Diapharma, West Chester Township, OH, USA) for FXa. To 100 µL *Vaa*SP-VX (diluted with PBS to final concentrations of 1 µM and 3 µM), 50 µL of Spectrozyme^®^, S-2288^TM^, S-2765^TM^, or S-2222^TM^ were added to final concentrations of 1 mM. Substrate hydrolysis was then examined on an Infinite M200 microplate reader (Tecan, Switzerland) by measuring A_405_.

Additionally, to test the FXa-like activity of *Vaa*SP-VX on prothrombin, we incubated 10 pmol of prothrombin with 20 pmol of *Vaa*SP-VX in 20 μL of a buffer consisting of 50 mM HEPES, 2 mM CaCl_2_, and 150 mM NaCl (pH 7.4). After 10 min of incubation at room temperature, the reaction mixture was 10-fold diluted with the buffer and transferred to a microplate well. After supplementing the reaction mixture with 10 μM α-thrombin-specific fluorogenic substrate Boc-Val-Pro-Arg-AFC (Peptide Institute Inc., Osaka, Japan), the hydrolysis of the substrate was assessed on an Infinite M1000 microplate reader (Tecan, Switzerland) by activating fluorescence at 380 nm and detecting it at 500 nm.

### 5.9. Testing of FIX and FX Activation

The activation of FIX and FX by *Vaa*SP-VX was determined using the following specific chromogenic substrates: Spectrozyme^®^ (American Diagnostics Inc., USA) for FIXa and S-2765^TM^ (Diapharma, USA) for FXa. In a well of a 96-well plate, 50 µL of 1 µM *Vaa*SP-VX was added to 50 µL of 100 nM FIX, and then incubated for 15 min at 37 °C. Furthermore, to 50 µL of 100 nM FX, 3 µM or even 10 µM *Vaa*SP-VX was added before the incubation. In negative controls, *Vaa*SP-VX was replaced with PBS. Activation of a particular blood coagulation factor by *Vaa*SP-VX was detected using an Infinite M200 microplate reader (Tecan, Switzerland). The activation of FIX or FX was detected via an increase in A_405_ following the addition of 50 µL of Spectrozyme^®^ (final concentration 1 mM) or S-2765^TM^ (final concentration 0.65 mM) to the reaction mixture, respectively.

### 5.10. Characterization of Proteolytic Specificity

The proteolytic activity of *Vaa*SP-VX was characterized by testing its ability to hydrolyze and/or activate blood coagulation factors and cofactors.

The fibrinogenolytic activity of *Vaa*SP-VX was examined as described in Sajevic et al. [43] using a human fibrinogen to *Vaa*SP-VX mass ratio of 100:1. It was then tested to determine whether the cleavage of fibrinogen by *Vaa*SP-VX resulted in the formation of a stable fibrin clot. To this end, 40 µL of *Vaa*SP-VX in PBS (0.1 to 10 µM final concentration), or 40 µL of thrombin (final concentration 1 µM; Helena Laboratories, USA) in a positive control, was added to a well of the 96-well microtiter plate. Subsequently, 40 µL of fibrinogen (final concentration 2 µM; Sigma-Aldrich, St. Louis, MO, USA) was added to a well and the fibrin clot formation was monitored at 405 nm (A_405_) using a microplate reader (Infinite M200, Tecan, Switzerland).

To examine the hydrolysis of FV and FX (Haematologic Technologies Inc., Essex Junction, VT, USA) by *Vaa*SP-VX, we used more easily accessible bovine FV, which appears to be functionally identical to human FV [19] and human FX. Seven micrograms of each substrate was incubated with or without (control) 1 μg of *Vaa*SP-VX in 25 µL of 20 mM Tris/HCl, 5 mM CaCl_2_, 140 mM NaCl, pH 7.5 at 37 °C. A 10 µL aliquot of the reaction mixture was withdrawn after 1, 3, 6, and 24 h, and analyzed using 12.5% (*m*/*v*) SDS-PAGE under reducing conditions. The gels were stained with PageBlue^®^ (Thermo Fisher Scientific, Vilnius, Lithuania). Protein bands were electroblotted from the gel to the PVDF membrane in Towbin transfer buffer (4 mM Tris/HCl, pH 7.5, 12 mM NaCl, and 20% (*v*/*v*) methanol) at 200 mA per gel for 90 min at room temperature. The PVDF membrane was immersed in 100% methanol for a few seconds, then moved to a 0.1% (*m*/*v*) Coomassie Blue R-250 (Thermo Fisher Scientific, Vilnius, Lithuania) + 1% (*v*/*v*) acetic acid + 40% (*v*/*v*) methanol solution for 1 min. The membrane was destained in 50% (*v*/*v*) methanol, then washed with water. Protein bands were excised and sequenced using automated Edman degradation on a Procise 492A Sequencing System (Applied Biosystems, Waltham, MH, USA).

### 5.11. Clotting of FVII-Deficient Plasma

Seventy-five microliters of commercial human plasma lacking FVII (HemosIL^®^, Lexington, MA, USA), 95 µL of 25 mM CaCl_2_ (Siemens, Germany), and 20 µL *Vaa*SP-VX in PBS to various final concentrations (refer to Figure 3C for concentrations) were individually pre-warmed to 37 °C. *Vaa*SP-VX (or only PBS in the control experiment) was then added to the plasma and incubated for 2 min at 37 °C, followed by the addition of 25 mM CaCl_2_ to induce clotting. Plasma clotting time was measured using the BFT II Coagulation Analyzer (Siemens, Germany).
